# Inaccessibility of care and inequitable conceptions of suffering: a collective response to the construction of “terminal” anorexia nervosa

**DOI:** 10.1186/s40337-023-00791-2

**Published:** 2023-05-02

**Authors:** Sam L. Sharpe, Marissa Adams, Emil K. Smith, Bek Urban, Scout Silverstein

**Affiliations:** Fighting Eating Disorders in Underrepresented Populations (FEDUP) Collective, 4400 North Congress Avenue Suite 100, West Palm Beach, FL 33407 USA

**Keywords:** Anorexia nervosa, Eating disorders, Lived experience, Peer support, Medical aid in dying, Standards of care, Iatrogenic harm

## Abstract

Informed by our lived experiences with eating disorders, our work providing direct support to communities underserved by existing healthcare structures, and our commitment to social justice, we are deeply troubled by several aspects of the proposed characteristics for “terminal” anorexia nervosa outlined by Gaudiani et al. in Journal of Eating Disorders (10:23, 2022). We have identified two substantial areas of concern in the proposed characteristics provided by Gaudiani et al. and the subsequent publication by Yager et al. (10:123, 2022). First, the original article and the subsequent publication fail to adequately address the widespread inaccessibility of eating disorder treatment, the lack of parameters for what constitutes “high quality care”, and the prevalence of trauma experienced in treatment settings for those who do access treatment. Second, the characteristics proposed for “terminal” anorexia nervosa are constructed largely based on subjective and inconsistent valuations of suffering which build on and contribute to harmful and inaccurate eating disorder stereotypes. Overall, we believe these proposed characteristics in their current form stand to detract from, rather than assist, the ability of patients and providers to make informed, compassionate, and patient-centered decisions about safety and autonomy both for individuals with enduring eating disorders and for individuals with more recently diagnosed eating disorders.

Gaudiani et al. [1] outlined a series of proposed clinical characteristics for “terminal” anorexia nervosa (AN) in J Eat Disord 10(1):23 2022 and provided three case studies from the Gaudiani Clinic as supporting evidence. These characteristics are: (1) a diagnosis of AN (2) age of 30 or older (3) prior persistent engagement in high-quality, multidisciplinary eating disorder (ED) care and (4) belief that further treatment will be futile and the patient’s acceptance of death in the context of established intact decision making capacity [1]. Several groups of clinicians published responses expressing their concerns with this proposed list of characteristics [2–4] to which Yager et al. responded with a comment published in J Eat Disord 10(1):135 2022 addressing these critiques and further arguing for the utilization of these characteristics for “terminal” AN [5]. Following the publication by Yager et al., Rosiel Elwyn responded in the Journal of Eating Disorders 11(1):2 2023 extensively detailing their perspective as an individual with living experience of severe and enduring anorexia nervosa^1^ (SE-AN) [6]. They emphasized caution related to the context, impacts, and potential for harm surrounding the proposed “terminal” AN characteristics. We share many of the concerns expressed in the preceding correspondences, and consider ours to be a novel and substantive continuation of their contributions. Our correspondence intends to contribute additional insight to challenge the construction of “terminal” AN, in particular regarding inequity in healthcare access and cultural narratives surrounding eating disorders (EDs). This response is informed by our own lived experiences with EDs and as providers of peer support, advocacy, and/or research and clinical practice in the United States (US).

## Defining high-quality, multidisciplinary eating disorder care

Our first concern is with characteristic (3), which necessitates “prior persistent engagement in high quality, multidisciplinary eating disorder care” [1, p. 11]. Yager et al. responded to the critique that this characteristic is poorly defined and ignores barriers to treatment access [4] by stating that “the proposed criteria require but do not presuppose the type or duration of high-quality eating disorder care to protect disadvantaged populations from the burden of diminished access to end-of-life care resources” [5, p. 6]. While not presupposing a specific type or duration of “high-quality eating disorder care”, the authors imply that such care exists and has been accessible for at least some amount of time for individuals with AN. The author’s framing of both treatment accessibility and quality [1, 5is discordant with our experiences, as patients, clinicians, and peer advocates, within systems of ED treatment. The authors [1, 5] fail to account for the interplay between treatment access, systemic oppression, iatrogenic harm, and conceptualizations of futility.

## Lack of accessible, high-quality, non-traumatic eating disorder treatment

Substantial and consequential differences in systems of ED care exist between countries, and a full comparison of their quality and accessibility is beyond the scope of this response. We will therefore focus on the context of the US, where the authors are based and where the three cases in the original manuscript [1] received treatment. Within the US landscape of ED treatment, an inpatient and/or residential program followed by progressive step-downs to lower levels of care is referenced by Yager et al. as the “optimal” treatment scenario that should be encouraged “wherever possible” [5, p. 7]. Concerningly, inpatient, residential, partial hospitalization, and intensive outpatient programs for EDs in the US (i.e. higher levels of care) are typically not individualized, are often administered within institutions controlled by private equity, and are beholden to insurance company’s interpretations of progress, which can impede an individual's full potential for healing [7–10]. While some therapeutic modalities utilized in ED treatment are evidence-based, there is a lack of comprehensive, impartial, and long-term research demonstrating the efficacy of the way these modalities are implemented in ED treatment settings^2^ [8, 10–13]. Less evidence still exists regarding the effectiveness of higher levels of care (HLOC) for treating members of communities that are underrepresented in ED care and ED research [9, 10, 14, 15]. The lack of documented treatment outcomes in the literature for those from underrepresented communities, calls into question the ability of ED treatment delivered within the US healthcare system to provide quality care to all individuals [16, 17]. This alone questions the validity of any diagnostic characteristics contingent upon the existence of such care.

Traumatic, coercive, and ill-suited experiences while in ED treatment are common [16, 18–21] and can directly produce the conditions that make continued attempts at recovery feel impossible and ineffective later in life, as occurred in case study 2 provided by Gaudiani et al. [1] and as was extensively detailed by Elwyn [6]. To an extent, ED treatment intentionally challenges tolerability as it includes changing behaviors and reframing cognitions that often develop as coping strategies to contend with trauma, oppression, and/or co-occurring mental and physical illnesses. Bias in clinician training, lack of necessary competencies, distressing milieu dynamics, and one-size-fits-all treatment modalities can result in treatment that is intolerable [16, 22] which impedes the potential for meaningful healing [23]. In their case presentations, Gaudiani et al. [1] recognize that the individuals’ traumatic and ineffective experiences in HLOC caused them to be unable to tolerate subsequent engagement in intensive treatment. However, they attribute these intolerances to the “highly sensitive” features of these individuals rather than acknowledging them as symptoms of systemic problems within the ED treatment field. While not treated in the US HLOC system, Elwyn describes how coercive and traumatic treatment experiences actively exacerbated the utility and foundational beliefs of their ED and directly produced subsequent unwillingness to engage in further treatment [6]. This is an example of iatrogenically-mediated progression of long-term EDs.

Elwyn [6] additionally highlights the circular logic Gaudiani et al. use to conceptualize characteristic (3).We share their  concern that characteristic (3) increases the likelihood that individuals who have not previously had access to “good quality” treatment will be labeled as “terminal” and therefore be less able to access quality treatment in the future, as the terminal prognosis would be seen to render further treatment futile [1, 6]. Yager et al. claims to advocate against setting a particular standard or duration for “high quality care” in order to “protect” [5, p. 6] underserved populations. However, rather than being protective, this facilitates a devaluation of marginalized lives by enabling their EDs to be deemed terminal and further treatment futile rather than holding treatment settings and systems of care accountable. This is especially consequential given that an estimated 8 out of 10 or more individuals with EDs never receive care at any level [24] so access to “a full course of treatment” is a privilege, not the norm. In the US, multi-disciplinary treatment is frequently prohibitively expensive– even for those who are insured, scholarships are rare, and discharges and step-downs are often mandated by insurance rather than medical and psychological readiness [25]. As a result, the “high-quality multidisciplinary” ED care endorsed by Yager et al. [5, p. 2] is not typical [8, 24, 26, 27]. Within the current treatment paradigm, access to expert ED care is severely limited and generally reserved for those with comprehensive insurance, geographic privilege, financial stability, low body weight, and medical acuity.

## Systemic oppression and barriers to care

Although the full relationship between systemic oppression, ED etiology, and healthcare inequity is beyond the scope of our correspondence, we will provide a brief description of barriers that impede marginalized and under-resourced people from accessing ED treatment that remain unaddressed by Gaudiani et al. [1] and Yager et al. [5] which underscore our concerns with criteria (3). First, those who do not have access to substantial wealth face barriers in accessing the range of ED care which is often medically indicated. Public insurance (i.e. Medicare and Medicaid) frequently do not cover treatment at many facilities that provide HLOC with step down care, leaving individuals who are under-resourced, including many of those who have long-term EDs, with limited treatment options beyond the inpatient level [8, 28, 29]. In our experience, ED outpatient providers who accept private insurance are also scarce, and those who see publicly insured patients are even fewer, and may have a cap mandated by the insurance provider on the number of sessions allotted, often regardless of illness severity. This reality renders access to ongoing treatment with “highly expert eating disorder providers” [1, p. 6] difficult or impossible for those who do not have comprehensive insurance or cannot pay out of pocket for care.

Access to care is also impacted by other identities and marginalization by systems of power. For example, ED diagnosis is typically delayed among people of color [24, 30] and there is a lack of culturally relevant ED care [31, 32], as ED treatment settings are overwhelmingly white, both visually and culturally. Disabled individuals may experience barriers to accessing treatment, including physically inaccessible facilities with insufficient space for mobility aids [33]. Patients with multiple chronic illnesses receiving ED treatment may encounter ED treatment staff who do not understand or account for their co-occurring chronic illnesses in the delivery of ED treatment [34, 35]. Programming and treatment modalities can be unwilling or unable to accommodate sensory and cognitive needs of neurodivergent people [22, 36]. This is particularly concerning as research has demonstrated that individuals with EDs may have increased rates of being on the autism spectrum [37–39]. Transgender and gender diverse people may be excluded from treatment centers due to sex and gender essentialist bathroom and/or roommate policies [40] despite the disproportionately high prevalence of EDs in this population [41–43]. Intersex individuals have long faced a medical paradigm that focuses on imposing medically unnecessary, and often traumatic, interventions in childhood [44, 45] and offers little competent medical care to intersex adults [46], including those with EDs. As weight bias is ubiquitous in society [47], healthcare [48], and ED treatment [49, 50], individuals with eating disorders who present at higher weights, including those with atypical anorexia nervosa (AAN), may experience dismissal or even encouragement of ED behaviors, may wait much longer to access treatment, tend to have enduring illness [51–53], and present with medical and psychological consequences similar to those with low-weight AN [54]. Individuals existing at the intersection of multiple marginalized identities may face additional unique and compounded barriers to receiving accessible and appropriate ED treatment [55]. Additionally, there is a general mistrust of healthcare systems among many underrepresented populations in ED treatment due to their unique histories of exploitation and discrimination by the medical system and accumulated experiences of receiving poor medical care [44, 56–58].

Eating disorders also significantly co-occur with other psychiatric disorders, as seen in the case reports [1], which are often exacerbated by continued malnutrition [2, 39, 59–61]. However, ED treatment providers may be under-resourced and unprepared to appropriately address co-occurring mental health concerns. Due to the complex entanglement of co-occurring disorders and EDs, other psychiatric disorders may interfere with remission of eating disorder symptomatology and negatively impact long-term healing if left untreated [62]. Overall, these factors contribute to treatment inaccessibility, avoidance of healthcare, and distrust of medical systems, which can become further entrenched by experiences in treatment that are more likely to be culturally incompatible, retraumatizing, and ill-suited for individuals within these populations.

As outlined above, there are multiple and intersecting forms of systemic oppression and lack of competencies that may prevent access to safe, affirming, and collaborative eating disorder care. Due to these factors, some individuals with a long standing ED may have never received specialized or intensive ED care or have received care which may, on paper, match the course of treatment described by Yager et al. as optimal but which may have done personal harm and contributed to the maintenance of the ED. Iatrogenic harm in the context of ED care can produce a vicious cycle where prior traumatic treatment may preclude future willingness to engage in treatment, making outpatient clinicians reluctant or unwilling to work with individuals who they deem too high risk [63]. In turn, this may increase the likelihood of involuntary treatment [2, 6, 61], contribute to further traumatic treatment experiences, increase the likelihood of clinical assessment of futility, exacerbate feelings of hopelessness, burdensomeness, and meaninglessness, and result in the potential entrenchment of these recurrences as inevitable [6]. We believe it is essential to evaluate, at every stage of an individual’s ED, whether true quality care has ever been previously accessible before engaging with conceptualizations of futility, treatment resistance, and terminal prognosis. Gaudiani et al. [1] critically overlook the inconsistent and unrealistic definition of characteristic (3), the failures of the US healthcare system to provide appropriate and accessible ED treatment for all individuals, the inadequate treatment of co-occurring disorders in ED care, and the potential of poor treatment itself to maintain or perpetuate the conditions facilitating the ED.

## The elusive and harmful definition of “terminal” anorexia nervosa

Our second concern is that the authors [1, 5] are ultimately presenting a largely affective definition of terminality, rather than one that is medically substantiated, which serves to further stereotypes about individuals with AN and reaffirm a harmful hierarchy of EDs. The authors’ assertion that AN can uniquely be considered to have a terminal stage due to its medical consequences, but that an individual does not need to exhibit medical consequences for their AN to be diagnosed as terminal, is unclear and contradictory [1, 5]. Gaudiani et al. states that “Anorexia nervosa is the only eating disorder that carries a guaranteed medical cause of death from malnutrition should weight loss continue unabated” with “a prognosis of less than 6 months” [1, p. 11]. The authors later propose that a “terminal” AN diagnosis is not based on “explicit physiologic markers or measurables” [1, p. 12] because “individuals should not be obliged to demonstrate extreme medical instability before having the right to choose to stop fighting” [1, p. 12]. Their inability to establish degrees of malnutrition which will inevitably result in death raises the question of why the medical complications of AN are necessary for its consideration as a proposed terminal illness, a designation which the authors argue cannot be applied to other psychiatric illnesses [5]. Additionally, the authors’ claim that only medical complications of AN warrant a right to palliative care and MAID perpetuates ED diagnosis hierarchies, as other types of EDs can and do result in substantial and potentially life-threatening medical complications [64].

## Shifting characteristics and discretionary application

While the authors defend characteristic (1) (a diagnosis of AN) and (2) (age 30 or older) based on evidence of increased mortality rates in individuals with long standing AN age 30 or older in the literature [1], they fail to provide adequate evidence that such individuals will be “guaranteed” to die from their AN within 6 months of ceasing recovery oriented behaviors. Although the authors acknowledge that “consensus regarding criteria for SE-AN remains elusive” [1, p. 2], they go on to describe “terminal” AN as a “subcategory of SE-AN” which constitutes a “distinct condition” [5, p. 2]. However, the lack of clinical consensus around characterizing “SE-AN” itself [62, 65, 66calls into question the claim that this category of ED or subcategories within it can be used to meaningfully assess predictable medical decline or mortality. Current definitions of SE-AN range from a minimum of 3 years to a minimum of 10 years of illness duration [67] and vary in whether they require previous “unsuccessful” treatment attempts and low body mass index (BMI) as qualifying criteria [68]. While a primary diagnosis of AN is generally considered a necessary component of SE-AN, this is complicated by the fact that ED diagnoses have overlapping criteria in the Diagnostic and Statistical Manual of Mental Health Disorders, fifth edition (text revision) (DSM-5-TR)[69] and many individuals with EDs may meet criteria for various eating disorders over the course of their lifetime. Multiple studies have found that crossover between anorexia nervosa restricting subtype (AN-R), AAN, anorexia nervosa binge-purge subtype (AN-BP), and/or bulimia nervosa (BN) over time is not uncommon [62, 70–76]. Notably, AN-R and AAN are distinguished only by BMI in DSM-5-TR, and AN-BP and BN are also distinguished only by BMI [69]. Clinical discretion in diagnosing ED patients on the “cusp” of meeting BMI criteria for AN further obfuscates these categories [51], with one study finding that minimal fluid shifts during sleep could result in divergent ED diagnoses from one day to the next [77]. How weight fluctuations or behavioral variability over the course of an enduring ED may impact eligibility for a diagnosis of SE-AN or “terminal” AN remains unclear, further complicating the application of these diagnostic categories.

In addition to the lack of clarity around defining SE-AN, multiple other factors may complicate staging criteria for AN. Given that deaths from medical complications of AN may be abrupt and unpredictable [78], some individuals live with AN for decades [79], and many individuals recover well after ten years of illness [2–4, 79–83], the claim that death will inevitably result within 6 months without active recovery work is unsubstantiated. As Gaudiani et al. [1, p. 4] points out, it is rare for individuals with AN to stop eating completely, and references from the original manuscript describe metabolic adaptation to caloric restriction in AN^84^] which can make it difficult to predict timelines of mortality and limits physiological comparisons to hunger strikes [85]. Additionally, individuals with AN are not homogenous and vary in their medical complications, co-occurring illnesses, types and intensity of ED behaviors, frequency of purging behaviors [64], and access to emergency care, resulting in variable mortality risks.

Despite the known overlap between ED categories and the heterogeneity within enduring AN, in a podcast interview, Gaudiani argued that while death is possible from EDs other than low weight AN, it cannot be considered inevitable within 6 months of ceasing treatment [86, 87]. Gaudiani further specifies that terminal AN criteria apply only to low weight AN due to her belief that people with AAN “seem to have a genetic capacity to spare certain systems including potentially becoming emaciated in a way that is more likely to be life protecting” [87], even at equivalent or more extreme levels of restriction than those with low-weight AN. This claim is uncited and in contrast to articles on the Gaudiani Clinic’s website and in Gaudiani's book claiming that AN and AAN are differentiated only due to weight bias [64, 88, 89], as well as existing research that has shown recent total weight loss, not BMI, is most closely associated with medical complications of AAN [54, 90].

The authors repeatedly state that the proposed characteristics and the possibility of MAID apply only to a small minority of individuals with long standing AN. However, we believe that Gaudiani et al. proposed characteristics and the subsequent comment by Yager et al. [5] have and will continue to have substantial and deleterious impacts beyond this population. Notably, the application of the “terminal” AN designation in all three case studies [1] to individuals who did not meet all proposed characteristics (one individual was weight restored when the switch to a palliative care approach was made and the other two had never completed a full course of treatment) indicates a diagnostic flexibility which may render a larger population than anticipated eligible to be labeled as “terminal.” This is additionally concerning given the active role played by the first author [1] in suggesting to her patients and their families the possibility of accepting that additional treatment would be “futile” [1, p. 4–10], and her suggestion in case 3 that a referral for MAID was “possible” [1, p. 8]. As Elwyn writes, this type of input can have a substantial “emotional and psychological impact on a person and their loved ones, and create a new experience in shaping how an individual thinks and relates to their experience, the feelings and responses of others, choices and outcomes” [6, p. 3].

## Reinforcing stereotypes and perceived diagnostic hierarchies

The publications by Gaudiani et al. and Yager et al. also reinforce the mis-conceptualization of individuals with long-term, low weight AN as not only medically but also constitutionally distinct from other individuals with EDs. Gaudiani et al. repeatedly described the case presentations as “rare,” “select,'” “highly sensitive,” “brilliant,” “brave,” and “incisive” and portrays the individual with “terminal” AN as possessing access to family support, quality treatment options, and consistent expert care. Collectively, the choice of these adjectives and these case studies furthers the deleterious and inaccurate stereotype of EDs as illnesses that affect sensitive, perfectionistic, and highly intelligent individuals of considerable privilege and relatively few economic or identity-based barriers to ED care.

The authors emphasize that both the case subjects and other hypothetical individuals meeting the proposed characteristics for “terminal” AN experience prolonged suffering, trauma from previous attempts at treatment, preference to avoid institutionalization with other ill individuals, extreme intolerance of weight gain, strong desire to have autonomy over their health care decisions, thoughts that future treatment is hopeless, and acceptance of inevitable death from their ED, which therefore precludes recovery oriented interventions [1, 5]. These elements of suffering and personal qualities are neither unique nor ubiquitous to individuals with low weight, enduring AN, but are present across the spectrum of different EDs at different body sizes, ages, and stages of illness [91–93]. The authors do not offer an analysis of how to contextualize similar factors in younger people with AN, except to say “nonetheless, the majority of patients with AN ultimately recover, and such expressions of anguish can be met with compassion and appropriate multidisciplinary care” [1, p. 12]. They also do not address how the values of compassion, autonomy, and harm reduction, which were critical in the decision to forgo involuntary treatment in the case studies, should be applied to commiserate suffering, exhaustion, and trauma experienced during the course of a higher weight or differently symptomatic ED.

Given the high rates of suicidality in EDs [15, 61, 94, 95], the comparable suffering experienced by many who do not meet characteristics (1) and (2), and the glorifying of the personal qualities of the case studies and hypothetical terminal AN patients, we are concerned that these characteristics may come to represent an aspiration for many who believe that their suffering and autonomy will only be respected if they can “succeed” [96] in maintaining and perpetuating their EDs sufficiently to reach characteristics (1) and (2). The belief held by some individuals with other types of EDs that being diagnosed with AN constitutes an honor, achievement, reward, or explicit goal is described in both academic and popular literature [97–104]. Within populations known to have a favorable orientation towards fulfilling AN diagnostic criteria, the likelihood that “terminal” AN will be perceived as additionally desirable by some individuals will likely be compounded by the fact that, in the case of “terminal” AN, the authors [1, 5] explicitly discard recovery oriented treatment, protocols, and mandates, which many patients find intolerable due to the excruciating difficulty of the healing process and the frequently traumatic nature of treatment settings. Although it may seem unfathomable that a diagnosis based on a terminal prognosis and acceptance of death would be perceived as desirable, co-occurring suicidality and major depressive disorder are common for individuals with EDs, as is direct or indirect engagement with one's ED as a method of death [105]. The ego-syntonic nature of some EDs may cause death from the disorder to feel more desirable than recovery and this may be further affirmed through iatrogenic harm, increases in ED identity, and loss of meaning and engagement with other aspects of life through repeated treatment admissions [6].

Finally, while the authors state that the proposed characteristics for terminal AN should not impact the vast majority of individuals with EDs [1], the promotion of “Terminal anorexia nervosa: three cases and proposed clinical characteristics” [1] on the Gaudiani Clinic’s public social media platforms [106, 107] has resulted in many individuals with current or past EDs, as well as their caregivers, being exposed to this controversial, consequential, and highly emotional consideration of how to assess and conceptualize the suffering and autonomy of individuals with EDs. We agree that repeatedly subjecting individuals to treatment they have previously experienced as ineffective and traumatic is harmful, and expanding other options beyond traditional treatment settings and the traditional full recovery paradigm must be a focus. We have also witnessed firsthand how the contents and distribution of this article have negatively impacted many vulnerable individuals (and their supporters) within our communities. This provides another example of how individuals with EDs are impacted by cultural conceptualizations of ED prognosis, orientation towards hopelessness, and deservingness of care.

## Conclusion

Gaudiani et al.'s framework for “terminal” AN assumes personal privilege, discounts systemic oppression, and fails to consider the interaction between diagnoses and perceived hierarchies across the full spectrum of EDs. The authors’ emphasis that terminal AN applies to “a select, rare group of adults” [5, p. 2] inevitably constructs a hierarchy of suffering that establishes a new type of eating disorder subject who uniquely deserves comfort and ultimately relief from their suffering. Consequently, this only adds to the inequalities and harms experienced across the spectrum of individuals with EDs and their loved ones, regardless of type of ED, age, and length of illness. Lastly, these characteristics have the potential to cause further dysfunction and inequity in the already dysfunctional and inequitable US healthcare system. We conclude by echoing Elwyn’s call to incorporate and prioritize those with lived and living experience, especially those harmed by and excluded from existing structures of care, in discussions of evolving diagnoses and protocols to better understand and support individuals with eating disorders.

## Data Availability

Not applicable.

## Abbreviations

AN: Anorexia nervosa
BN: Bulimia nervosa
AAN: Atypical anorexia nervosa
SE-AN: Severe and enduring anorexia nervosa
ED: Eating disorder
EDs: Eating disorders
US: United States
HLOC: Higher levels of care
BMI: Body mass index
MAID: Medical aid in dying
DSM-5-TR: Diagnostic and Statistical Manual of Mental Health Disorders, fifth edition, text revision
AN-R: Anorexia nervosa, restricting subtype
AN-BP: Anorexia nervosa, binge-purge subtype

## Footnotes

1 We will use the term severe and enduring anorexia nervosa (SE-AN) in this correspondence only when referencing the work and experience of other people out of respect for their self-identification. We are generally uncertain about the utility and benefit of this term, as it is poorly defined, lacks consensus, and can deleteriously impact self-perception of eating disorder patients. This categorical separation of a longer-term subset of anorexia nervosa from other eating disorders can contribute to increased hopelessness in individuals classified as SE-AN and imply to others that their eating disorder experiences are not severe, reaffirming “not sick enough” narratives. We find longer-term, long standing, or enduring eating disorders to be more appropriate and adopt their use in this correspondence.

2 Much of the existing research on the efficacy of HLOC treatment in the US consists of short-term studies conducted by treatment centers on their own patient outcomes. This introduces potential bias due to author affiliations with the treatment facilities under evaluation, non-representative populations due to treatment inaccessibility, no control groups, and frequently no follow-ups beyond 6 months post discharge.
